# Role of Amphipathic Helix of a Herpesviral Protein in Membrane Deformation and T Cell Receptor Downregulation

**DOI:** 10.1371/journal.ppat.1000209

**Published:** 2008-11-21

**Authors:** Chan-Ki Min, Sun-Young Bang, Bon-A Cho, Yun-Hui Choi, Jae-Seong Yang, Sun-Hwa Lee, Seung-Yong Seong, Ki Woo Kim, Sanguk Kim, Jae Ung Jung, Myung-Sik Choi, Ik-Sang Kim, Nam-Hyuk Cho

**Affiliations:** 1 Department of Microbiology and Immunology, College of Medicine and Institute of Endemic Diseases, Seoul National University Medical Research Center and Bundang Hospital, Seoul, Korea; 2 Department of Life Science and School of Interdisciplinary Bioscience and Bioengineering, Pohang University of Science and Technology, Pohang, Kyungbuk, Korea; 3 Seoul National University Hospital, Innovative Research Institute for Cell Therapy, Chongno-Gu, Seoul, Korea; 4 National Instrumentation Center for Environmental Management, Seoul National University, Gwanak-Gu, Seoul, Korea; 5 Department of Molecular Microbiology and Immunology, University of Southern California School of Medicine, Los Angeles, California, United States of America; Oregon Health & Science University, United States of America

## Abstract

Lipid rafts are membrane microdomains that function as platforms for signal transduction and membrane trafficking. Tyrosine kinase interacting protein (Tip) of T lymphotropic Herpesvirus saimiri (HVS) is targeted to lipid rafts in T cells and downregulates TCR and CD4 surface expression. Here, we report that the membrane-proximal amphipathic helix preceding Tip's transmembrane (TM) domain mediates lipid raft localization and membrane deformation. In turn, this motif directs Tip's lysosomal trafficking and selective TCR downregulation. The amphipathic helix binds to the negatively charged lipids and induces liposome tubulation, the TM domain mediates oligomerization, and cooperation of the membrane-proximal helix with the TM domain is sufficient for localization to lipid rafts and lysosomal compartments, especially the mutivesicular bodies. These findings suggest that the membrane-proximal amphipathic helix and TM domain provide HVS Tip with the unique ability to deform the cellular membranes in lipid rafts and to downregulate TCRs potentially through MVB formation.

## Author Summary

Herpesvirus persists in its host by entering a latent state, periodically reactivating to produce infectious viral particles. Some of the herpesviruses have also been known to be related to cancers. Herpesvirus saimiri (HVS), an oncogenic monkey herpesvirus, persists in the T lymphocytes of its natural host, the squirrel monkey, without any apparent disease symptoms, but infection of other species of New World and Old World primates results in fulminant T cell lymphomas. Two viral oncoproteins, Saimiri Transforming Protein and Tyrosine kinase-interacting protein (Tip), are required for T cell transformation. It has been known that Tip may also play some role in viral persistency within T cells by inhibiting the activation of the host cells upon antigenic stimulation. Here, we have identified a structural domain, a putative amphipathic helical motif, preceding the transmembrane domain of Tip. We also found that the structural motif is essential for Tip's localization on specialized membrane domains, lipid rafts, and selective downregulation of antigen receptors. Furthermore, we could genetically dissect the functional roles of the amphipathic helical motif and transmembrane domain of Tip in membrane deformation and oligomerization, respectively. These findings significantly advanced our understanding of how herpesvirus modulates host lymphocytes for viral persistence and pathogenesis.

## Introduction

Lipid rafts are membrane microdomains that take part in coordinating cell signalling functions and membrane trafficking. In T cells, upon antigenic stimulation, T cell receptors (TCRs) are recruited to lipid rafts, where they transmit signals via several pathways. The TCR signals induce the anchoring of lipid rafts to the underlying actin cytoskeleton, resulting in the assembly of lipid rafts [1]. Subsequently, clustered lipid rafts, containing TCR/CD3 complexes, are subjected to endocytosis, and the TCR/CD3 complexes are targeted for lysosomal degradation [2]. Thus, current evidence indicates that lipid rafts function as platforms for both the signalling and endocytosis of activated TCRs. Despite the important role of lipid rafts in signalling and membrane trafficking in T cells, the regulatory mechanisms controlling membrane trafficking to lysosomal compartments remain unclear. Several biochemically distinct compartments for membrane trafficking have been identified in other cell types including primary endocytic vesicles, early endosomes, late endosomes, and lysosomes. It has been recently demonstrated that multivesicular bodies (MVB), also known as vesiculated late endosomes, are required for many key trafficking processes such as the downregulation of activated signalling receptors [3]. However, difficulties in elucidating the mechanisms of membrane trafficking have been compounded in T cells, because the fate of endocytic vesicles and the dynamics of transport intermediates remain uncertain.

Herpesvirus persists in its host by entering a latent state, periodically reactivating to produce infectious viral particles. Herpesvirus saimiri (HVS), an oncogenic γ2 herpesvirus, persists in the T lymphocytes of its natural host, the squirrel monkey, without any apparent disease symptoms, but infection of other species of New World and Old World primates results in fulminant T cell lymphomas [4]. In addition, when HVS infects the primary T lymphocytes of humans, Old World primates, New World primates, or rabbits, it can immortalize infected T cells, allowing them to grow independently of IL-2 [5].

Tyrosine kinase-interacting protein (Tip) is encoded in the first open reading frame at the left end of the highly oncogenic strains of HVS. Tip is not required for viral replication, but is required for T cell transformation in cultures, and for lymphoma induction in primates [4]. Tip has multiple binding sites for cellular proteins. The interaction of Tip with Lck kinase, which is mediated by the Src homology 3-binding (SH3B) motif and C-terminal Src-related kinase homology (CSKH) domain of Tip [6],[7], interferes with early events in the TCR signal transduction pathway, resulting in inhibition of immunological synapse formation [8]. Tip also interacts with p80, a novel cellular endosomal protein that contains an N-terminal WD repeat domain and a C-terminal coiled-coil domain [9]. The interaction of Tip with p80, which is mediated by a region containing a serine-rich (SR) motif, facilitates the formation of enlarged lysosomal vesicles, and results in the targeting of Lck and TCR/CD3 complexes for lysosomal degradation. We have previously demonstrated that Tip constitutively localizes in lipid rafts and exploits Lck and p80 to recruit TCR/CD3 complexes, leading to lipid raft aggregation and internalization [10]. Constitutive localization of Tip in lipid rafts depends on the C-terminal transmembrane (TM) domain, but not Lck and p80 interaction, and is also necessary for the efficient downregulation of TCR/CD3 and CD4 surface expression without affecting the inhibition of TCR signal transduction [11].

In this study, we report the presence of a putative amphipathic helical motif preceding the TM domain of Tip. Structural analysis revealed that Tip's amphipathic helical motif is composed of hydrophobic and positively-charged amino acid residues. Recently, the amphipathic helical motif has attracted much attention due to its active role in membrane curvature formation and membrane trafficking [12]. Thus, we investigated roles of the amphipathic helical motif in the molecular functions of Tip, including lipid raft localization and downregulation of TCR/CD3 and CD4. We found that the membrane-proximal amphipathic helical motif is required for the efficient localization of Tip in lipid rafts as well as its selective downregulation of TCR/CD3, potentially through deformation of membrane structures and MVB formation in T cells.

## Results

### The membrane-proximal cytoplasmic domain of Tip is required for efficient association with lipid rafts

**Figure 1:**
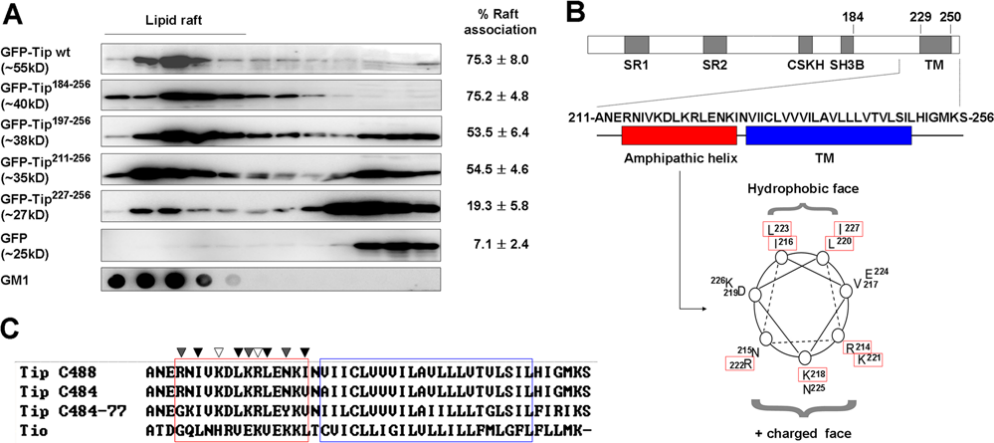
Tip's membrane-proximal amphipathic helix is required for efficient association with lipid rafts. (A) Lipid raft association of Tip or its deletion mutant. 293T cells were transfected with plasmids encoding Tip or its deletion mutants as GFP fusion proteins, and processed for lipid raft fractionation. Proteins from each fraction of the sucrose gradient were subjected to immunoblotting with an anti-GFP antibody to detect Tip or its mutants. CTB-HRP was used to confirm the localization and integrity of the lipid rafts. The degree of lipid raft association was estimated by densitometry analysis of triplicate samples and is indicated as the average percentage value (±S.D.) of raft association in the right side of each panel. (B) Schematic diagram of Tip protein and the structure of a membrane proximal amphipathic helix. SR, serine-rich; CSKH, C-terminal Src-related kinase homology; SH3B, Src homology 3-binding; TM, transmembrane domain. (C) Conservation of the amino acid sequences of the amphipathic helix (red box) in Tip of Herpesvirus saimiri strains and in Tio of Herpesvirus ateles. White triangle, conserved positive charges; gray triangle, moderately-conserved positive charges; black triangle, conserved hydrophobic residues.

We have recently reported that the TM domain (amino acid residues 229–250) of Tip is required for its association with lipid rafts, while other motifs involved in interactions with Lck and p80 are dispensable for lipid raft targeting [11]. In this study, GFP-Tip fusion proteins carrying deletions from the cytoplasmic region of Tip were generated (Figure S1), and the motif required for lipid raft localization was mapped. The degree to which Tip was associated with lipid rafts, in 293T cells, was estimated by densitometry and represented as the average percentage value from triplicate samples. The position and the integrity of lipid rafts in the discontinuous sucrose gradient were determined by the presence of GM1 ganglioside, which associates reliably with lipid rafts (Figure 1A). We found that the wild type GFP-Tip fusion protein was efficiently associated with lipid rafts (approximately 75%), which is consistent with our previous results [10],[11]. Deletion of Tip's cytoplasmic domain, which contains known protein interaction motifs, had no discernible effect on the association of Tip with lipid rafts (GFP-Tip^184-256^). Two additional deletion mutants, GFP-Tip^197-256^ and GFP-Tip^211-256^, showed a similar Tip distribution; approximately 50% was associated with lipid rafts. A GFP-Tip^227-256^ mutant carrying only the Tip TM domain was detected primarily in the fractions where cytoplasmic GFP protein localized, and showed only 20% association with lipid rafts. These results suggest that the C-terminal cytoplasmic regions proximal to the TM domain might contribute significantly to Tip's localization to lipid rafts.

To exclude the potential confounding effects by GFP fusion on lipid raft association of Tip, we also constructed flag-tagged version of Tip mutants and examined their localization on lipid rafts (Figure S2). In this independent experiment, similar level of lipid raft association of flag-tagged Tip mutants was observed when compared with those of GFP-fusion proteins, indicating that GFP fusion does not significantly affect the lipid raft association of Tip and its mutants.

We next analyzed the potential structure of the C-terminal regions spanning residues 184 to 256 of Tip using a protein structure prediction server [13]. The secondary structure analysis predicted with high confidence that the amino acid residues from 213 to 250 of Tip would form an α-helix (Figure S3). Notably, the amino acid residues from 213 to 228, proximal to the TM domain, are composed of hydrophobic and positively-charged amino acid residues, and are predicted to form an amphipathic helical structure (Figure 1B). This amphipathic helical motif was highly conserved in Tip from three different strains of HVS, and in Tio (Two-in-one) of Herpesvirus ateles (HVA), a recently identified member of the γ2-herpesvirus family (Figure 1C and Figure S3). Tio, an oncoprotein of HVA, has been shown to induce transformation of T cells in a manner similar to that seen in StpC and Tip of the C488 strain of HVS [14]. These findings suggest that this potential amphipathic helical motif preceding the TM domain might play a role in Tip function.

### The amphipathicity of the membrane-proximal helix is required for the efficient association of Tip with lipid rafts

**Figure 2:**
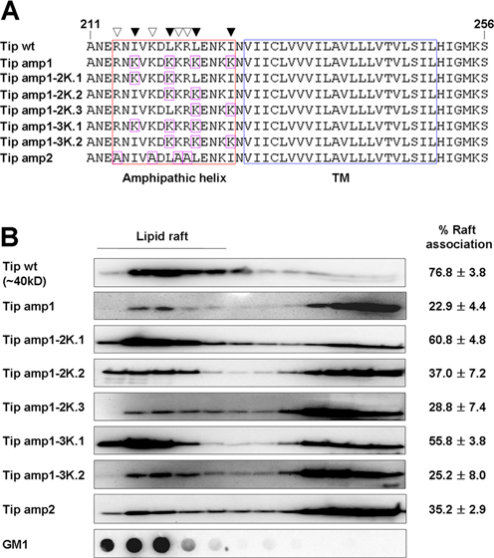
Amphipathicity of the membrane-proximal helix is required for efficient association with lipid rafts. (A) Point mutations in the amphipathic helical region of Tip. Mutated amino acids are highlighted as purple squares within the helical region (red box). White triangle, amino acids in positively charged face; black triangle, amino acids in hydrophobic face. (B) Lipid raft association of Tip or its mutants. The relative association of Tip or its mutants with lipid rafts was analyzed as described in Figure 1. The degree of lipid raft association was estimated by densitometry analysis and indicated as percentage raft association in the right side of each panel.

To evaluate the effect of the amphipathicity of Tip's membrane-proximal helical motif upon its lipid raft localization, this motif was mutated by replacing the hydrophobic and charged residues with lysine and alanine, respectively (Figure 2A). The resulting ability of the Tip mutants to associate with lipid rafts was then assessed. When Tip's four conserved hydrophobic residues (I^216^, L^220^, L^223^, and I^227^), predicted to form hydrophobic face, were replaced with lysines (Tip amp1), there was a ∼50% reduction in lipid raft association, in comparison to wild type Tip (Figure 2B). The degree of lipid raft association observed in this mutant was similar to that observed in the GFP-Tip^227-256^ mutant, carrying only the TM domain of Tip (Figure 1A). As such, these data suggest that the four conserved hydrophobic residues are critical for targeting of Tip to the lipid rafts. Sequential replacement of two or three consecutive, topologically-adjacent hydrophobic residues with lysine residues (Tip amp1-2K.1∼Tip amp1-3K.2) resulted in a gradual reduction in Tip's lipid raft association, ranging from 16% to 54% of the degree of association observed in wild type Tip. Both Tip amp1-2K.3 and Tip amp1-3K.2 mutants carrying lysines proximal to the TM domain showed a low degree of lipid raft association, similar to that observed in Tip amp1, indicating that the hydrophobic isoleucine and leucine residues proximal to the TM domain are more critical for the association with lipid rafts than are the more distal residues. Substitution of the positively-charged residues with alanine (Tip amp2: R^214^, K^218^, K^221^, and R^222^) also resulted in a ∼40% reduction in lipid raft association compared to wild type, demonstrating the significant contribution of these residues to Tip's localization to lipid rafts.

### Amphipathicity of the membrane-proximal helix is required for Tip's downregulation of TCR/CD3 surface expression, but not for CD4

**Figure 3:**
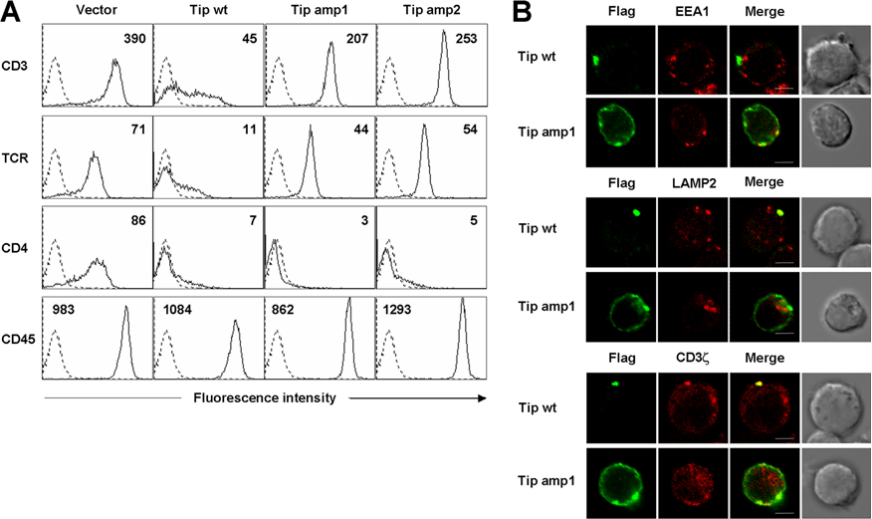
Downregulation of TCR/CD3 by Tip or its mutants. (A) Jurkat T cells were transfected with pBabe, pBabe/Tip, pBabe/Tip amp1, or pBabe/Tip amp2 and selected with puromycin (5 µg/ml). Jurkat T cells stably expressing Tip or its mutants were then analyzed by flow cytometry to detect the level of surface expression of TCR, CD3, CD4, and CD45. Mean fluorescence intensities were noted in each box. Dotted line, isotype control. (B) Jurkat T cells transiently expressing Flag-tagged wild type Tip or Tip amp1 were fixed and stained with an anti-Flag antibody (green) together with anti-EEA1, an early endosomal marker, anti-LAMP2, a late endosomal or lysosomal marker, or anti-CD3ζ antibodies (red).

Tip-mediated downregulation of TCR/CD3 and CD4 depends on its ability to associate with lipid rafts [11]. To examine the contribution of amphipathicity of Tip's membrane-proximal helix to this downregulation, levels of TCR, CD3, CD4, and CD45 surface expression were examined in Jurkat T cells stably expressing wild type Tip, Tip amp1, or Tip amp2, using flow cytometry. As shown previously [9], expression of wild type Tip in T cells effectively downregulated the surface expression of TCR/CD3 and CD4 (Figure 3A). In striking contrast, the downregulation of TCR and CD3 surface expression was severely impaired in Jurkat T cells expressing Tip amp1 or Tip amp2 (Figure 3A). However, downregulation of CD4 was not significantly affected by the mutations which abolished the amphipathicity of the membrane-proximal helix. Neither wild type Tip nor its mutants had any significant effect upon the surface expression of CD45, demonstrating the specificity of Tip's effects for TCR and CD3 downregulation. These results indicate that the amphipathicity of Tip's membrane-proximal helix is involved in the downregulation of TCR/CD3, but not CD4 surface expression.

We have previously shown that Tip's targeting to the lysosomal compartments involves its formation of a complex containing Lck and p80 [9]. Tip's formation of this complex is correlated with its lipid raft association and the lysosomal degradation of TCR/CD3 complexes [10],[11]. To examine whether loss of amphipathicity in Tip's membrane-proximal helix might affect the lysosomal localization of the viral proteins and TCR/CD3, Jurkat T cells transiently expressing Tip or Tip amp1 were reacted with antibodies specific to EEA1, an early endosomal marker, LAMP2, a late endosomal/lysosomal marker, or CD3ζ and then examined under a confocal microscope (Figure 3B). To quantitatively compare the degree of colocalization of the proteins in the vesicular compartments, we measured the Pearson correlation coefficient (R) values (see Materials and Methods) for each set of colocalizing proteins in 10 to 20 cells (Figure S4). Vesicles containing wild type Tip were weakly colocalized with EEA1 (average R value = 0.16), but were strongly colocalized with LAMP2 (R = 0.76) or CD3ζ (R = 0.82), as shown previously [10]. In contrast, Tip amp1 displayed partial colocalization with EEA1 (R = 0.51) and CD3ζ (R = 0.60) but did not colocalize with LAMP2 (R = 0.02), indicating that the amphipathicity of Tip's membrane-proximal helix is required for efficient lysosomal targeting of Tip and TCR/CD3.

### Tip^211-256^ is sufficient for lysosomal localization and can be detected in membrane curvatures and MVBs of vesicular compartments

**Figure 4:**
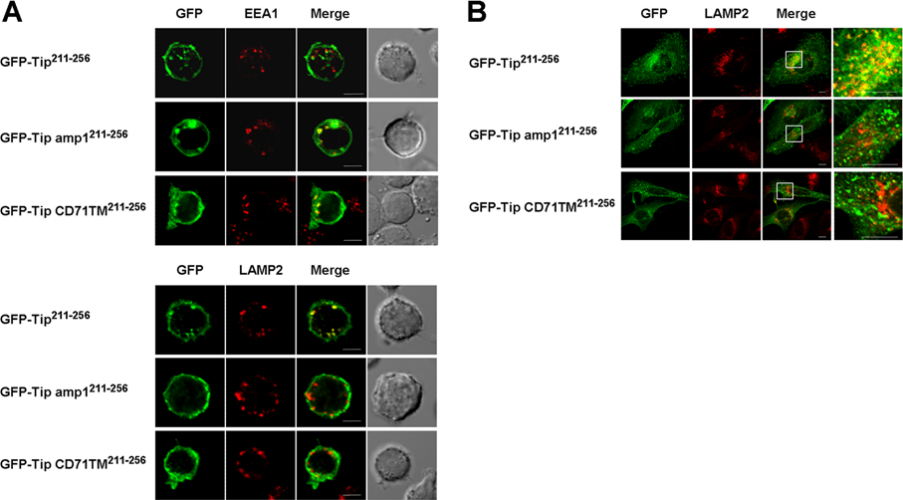
Localization of Tip^211-256^ in lysosomal compartments. (A) Jurkat T cells transiently expressing GFP-Tip^211-256^, GFP-Tip amp1^211-256^, or GFP-TipCD71TM^211-256^ (green) were reacted with antibodies specific to EEA1 (red, upper panel), or LAMP2 (red, lower panel). Merged images show colocalization of the GFP fusion proteins with EEA1 or LAMP2. Bar, 5 µm. (B) HeLa cells were transfected with plasmids encoding GFP-Tip^211-256^, GFP-Tip amp1^211-256^, or GFP-TipCD71TM^211-256^ (green) and subsequently analysed in an immunofluorescence assay, using an anti-LAMP2 antibody (red). The amplified image within the white rectangle of the merged image is shown on the right. Bar, 5 µm.

We have previously shown that Tip's TM domain is required for its lysosomal trafficking [11]. To investigate the role of the membrane-proximal amphipathic helix and TM domain in Tip's lysosomal localization, Jurkat T cells expressing GFP-Tip^211-256^, GFP-Tip amp1^211-256^, or GFP-Tip CD71TM^211-256^ were reacted with antibodies specific to EEA1 or LAMP2 (Figure 4A). Colocalization of the fusion proteins with the endocytic markers were evaluated quantitatively for Pearson correlation coefficient (Figure S5). The intracellular vesicles containing GFP-Tip^211-256^ colocalized strongly with LAMP2 (R = 0.82), but weakly with EEA1 (R = 0.21), suggesting that the amphipathic helix and TM domain are sufficient for Tip fusion proteins to be delivered into the late endosomes or lysosomes. In fact, almost all of the intracellular vesicles containing GFP-Tip^211-256^ were costained with LAMP2 in the transfected T cells (data not shown). Unlike GFP-Tip^211-256^, both GFP-Tip amp1^211-256^ and GFP-Tip CD71TM^211-256^, a Tip mutant carrying the TM domain of CD71 in place of that of Tip (Figure S1), were only partially localized in LAMP2-positive vesicles, showing slight preferential colocalization with EEA1 (R values range from 0.34 to 0.46, Figure 4A and S5A). These results were further confirmed in HeLa cells expressing these fusion proteins (Figure 4B and S5B). The colocalization of lysosomes with vesicles containing GFP-Tip^211-256^ (R = 0.54) was also more prominent than with vesicles containing GFP-Tip amp1^211-256^ (R = 0.30) or GFP-TipCD71TM^211-256^ (R = 0.37), in HeLa cells.

**Figure 5:**
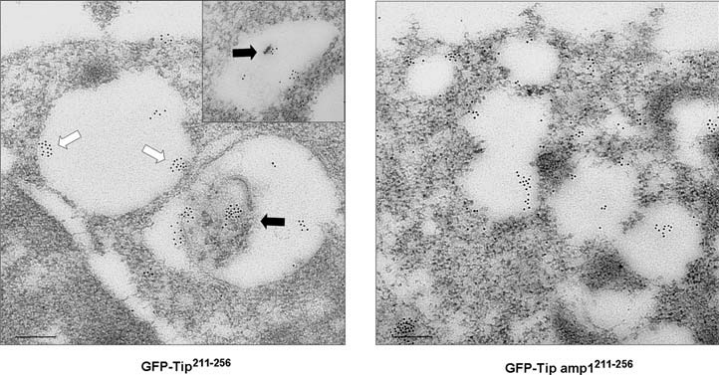
Ultrastructural identification of GFP-Tip^211-256^- or GFP-Tip amp1^211-256^-containing vesicles. Jurkat T cells transiently expressing GFP-Tip^211-256^ or GFP-Tip amp1^211-256^ were examined by immunoelectron microscopy. The GFP fusion proteins were detected with an anti-GFP primary antibody and an anti-rabbit IgG secondary antibody conjugated with 10 nm gold particles. The proteins were localized in membrane curvatures (white arrow) or in membranous complexes within the lumen (black arrow) in endocytic vesicles. Bar, 0.2 µm.

To determine in more detail the distribution of the GFP fusion proteins, Jurkat T cells expressing GFP-Tip^211-256^ or GFP-Tip amp1^211-256^ were analyzed by immunoelectron microscopy after staining with gold-conjugated anti-GFP antibodies. The gold signal was generally associated with intracellular vesicular compartments. Interestingly, GFP-Tip^211-256^ was frequently detected in luminal buddings of vesicular membranes, or in membranous complexes within the lumen (Figure 5), reminiscent of the process of MVB formation [15]. MVBs form by budding into the lumen of the vacuolar endosomes, which carry membrane proteins selected for the late endosomal route. They are thought to fuse with late endosomes or, following maturation, directly with lysosomes [15]. In cells expressing GFP-Tip amp1^211-256^, GFP was generally associated with smaller vesicular compartments, most likely the early endosomes, as shown in Figure 4. Associations of membrane curvature or MVBs with the fusion proteins were barely detectable (Figure 5). Taken together, it appears that the peptide encompassing the membrane-proximal helix and the TM domain of Tip might be involved in MVB formation in late endosomal compartments where Tip and its complex are degraded.

### The membrane-proximal amphipathic helix of Tip binds to negatively charged lipids and induces liposome deformation

**Figure 6:**
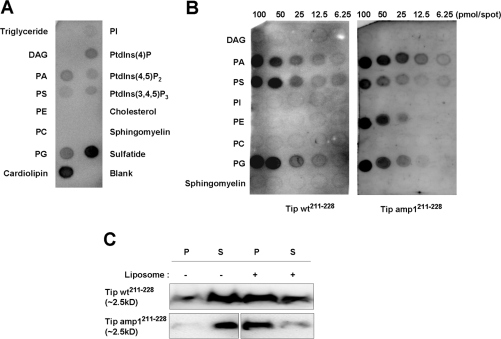
The amphipathic helical peptides of Tip interact directly with acidic lipids and liposomes. (A) Synthetic peptides derived from Tip's amphipathic helical region (amino acids 211-228) were incubated with a lipid array. The biotin-labeled peptides (0.4 µM) were incubated overnight at 4°C and were subsequently probed with streptavidin-HRP-conjugated antibodies. DAG, diacylglycerol; PA, phosphatidic acid; PS, phosphatidylserine; PE, phosphatidylethanolamine; PC, phosphatidylcholine; PG, phosphatidylglycerol; PI, phosphatidylinositol; PtdIns, phosphatidylinositides. (B) Peptides derived from wild type Tip or Tip amp1 were used to probe a lipid array, with decreasing concentrations from 100 to 6.2 pmol, to assess binding specificity. Peptides (0.4 µM) showed saturated binding at ∼50 pmol of certain lipids (see also Figure S4). (C) The peptides were incubated in the presence or absence of liposomes composed of PC (65% mol/mol), PS (25% mol/mol), and cholesterol (10% mol/mol) at room temperature for 10 min. The peptide-liposome complexes were sedimented by ultracentrifugation and subsequently resolved by SDS-PAGE to detect binding peptides. P, pellet; S, supernatant.

Membrane curvature is an active means for creating membrane domains and organizing trafficking [12]. Several mechanisms have been suggested to constitute active cellular processes for the formation of membrane curvature, and these include changes in lipid composition, oligomerization of curvature scaffolding proteins, and the insertion of amphipathic helices into the lipid bilayer [12],[16]. The possibility that the membrane-proximal amphipathic helix of Tip interacts with lipids was examined using a lipid binding assay. As shown in Figure 6A, a synthetic peptide derived from the membrane-proximal amphipathic helix of Tip (Tip wt^211-228^) was found to bind to a series of negatively charged lipids including phosphatidic acid (PA), phosphatidylserine (PS), phosphatidylglycerol (PG), cardiolipin, phosphatidylinositide (PtdIns), and sulfatide, but did not bind to other neutral or positively-charged lipids such as triglyceride (TG), diacylglycerol (DAG), phosphatidylenthanolamine (PE), phosphatidylcholine (PC), cholesterol, or sphingomyelin. The binding specificity of the peptide to these lipids was further examined by probing an array of several lipids immobilized on nitrocellulose membranes at concentrations ranging from 100 pmol to 6.2 pmol. As demonstrated in Figure 6B, both wild type (Tip wt^211-228^) and mutant (Tip amp1^211-228^) peptides, carrying lysine residues instead of hydrophobic amino acids, were able to bind dose-dependently to PA, PS, and PG, with binding saturation occurring at approximately 50 pmol of lipids (Figure 6B and Figure S6). Interestingly, the mutant peptides derived from Tip amp1^211-228^ were also capable of binding to PE. The peptide-lipid interactions were further validated in a liposome binding assay, in which liposomes composed of 65% PC, 25% PS and 10% cholesterol were reacted with biotin-conjugated peptides, then cosedimented by ultracentrifugation. The coprecipitated peptides were resolved by gel electrophoresis and were subsequently probed with streptavidin-HRP conjugates. As shown in Figure 6C, approximately 50% of Tip wt^211-228^ peptides were cosedimented with liposomes, whereas less than 5% precipitated in the absence of liposomes. The Tip amp1^211-228^ peptides also precipitated after incubation with liposomes, even more efficiently than did the amphipathic wild type peptide. These data suggest that the hydrophobic residues of the amphipathic helix had little effect upon peptide-lipid binding properties. It remains a possibility, however, that these residues might restrict the affinity and preference of Tip for specific lipids.

**Figure 7:**
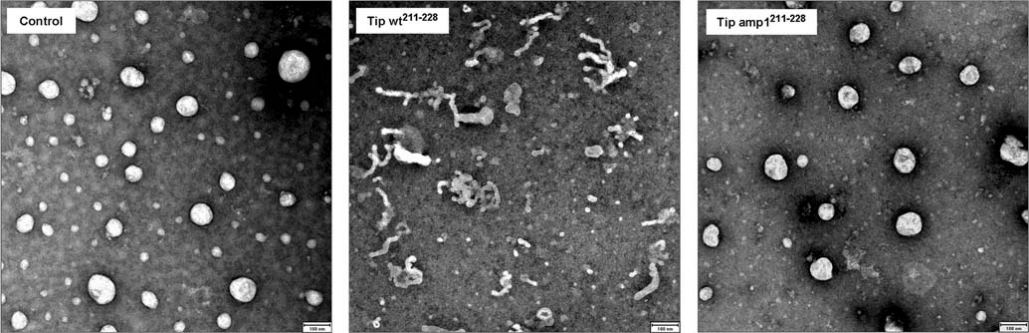
Liposome tubulation by the amphipathic helical peptide Tip wt^211-228^. Liposomes were incubated with peptides (4 µM) at room temperature for 10 min, stained, and examined by electron microscopy. Tubulation was not observed in the absence of peptide (control) or in the presence of mutant helical peptides (Tip amp1^211-228^), in which hydrophobic residues were replaced with lysines. Bar, 100 nm.

The influence of the amphipathic helix upon membrane curvature formation was examined using a liposome-based membrane deformation assay [17],[18],[19]. The peptides were incubated with liposomes, which have the same lipid composition as those used in Figure 6, and subsequently examined by electron microscopy (Figure 7). Tip wt^211-228^ peptides resulted in efficient and robust formation of tubules with diameters of 20–40 nm, whereas Tip amp1^211-228^ did not. A laser light scattering assay revealed that incubation with Tip wt^211-228^ peptides dramatically altered the size distribution of the liposomes into ranges of 40–250 nm, whereas no significant changes in liposome size were detected following incubation with Tip amp1^211-228^ (Figure 7 and Figure S7).

Collectively, the reported results indicate that the positively-charged residues within Tip's amphipathic helix confer a specific affinity for negatively-charged phospholipids, potentially through ionic interactions. The conserved hydrophobic residues enable the amphipathic helix to act like a wedge inserted into the membrane, to induce membrane curvature.

### Tip is oligomerized through its TM

**Figure 8:**
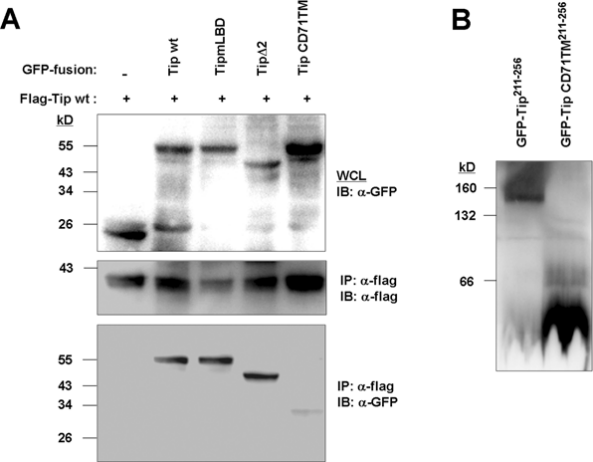
Oligomerization of Tip. (A) 293T cells were transfected with plasmids encoding Flag-tagged wild type Tip and GFP-fusion proteins of wild type Tip or its mutants. Interactions between the proteins were analyzed using immunoprecipitation with anti-Flag antibodies and subsequent immunoblotting with anti-GFP or anti-Flag antibodies. (B) 293T cells transiently expressing GFP-Tip^211-256^ or GFP-TipCD71TM^211-256^ fusion proteins were resolved by Blue Native PAGE analysis, and fusion proteins were detected using an anti-GFP antibody.

Previously, we demonstrated that Tip can induce the aggregation of lipid rafts and enhance the recruitment of lipid raft-resident proteins, eventually forming large vesicular compartments in T cells [9],[11]. These results suggested that Tip might oligomerize within membrane microdomains, inducing structural changes in the lipid bilayer. As such, the possibility of Tip oligomerization was investigated by coexpressing flag-tagged wild type Tip and GFP-Tip fusion proteins, then immunoprecipitating with an anti-flag antibody (Figure 8A). Immunoblotting with an anti-GFP antibody revealed that the flag-tagged Tip co-precipitated with the GFP-Tip fusion protein, but not with GFP, suggesting that Tip interacts with itself. To determine the region responsible for Tip oligomerization, GFP-Tip mutants were included in the immunoprecipitation assay [11]. GFP-Tip mutants no longer binding with Lck (TipmLBD) or p80 (TipΔ2) formed an immune complex with flag-tagged wild type Tip, indicating that Tip's Lck- and p80-binding motifs do not participate in Tip oligomerization. However, a Tip mutant carrying the TM domain of CD71 (Tip CD71TM) in place of its native one failed to interact with wild type Tip, suggesting that Tip's TM domain mediates its oligomerization.

Recently, Mitchell et al., reported that Tip is present as monomeric form in solution based on hydrogen-exchange mass spectrometry and circular dichroism study [20]. However, the recombinant protein they used contains only cytoplasmic region without transmembrane domain of Tip. Thus, our current result using the full-length Tip protein including transmembrane domain is more appropriate in reflecting natural status of Tip *in vivo*.

Oligomerization of Tip was further confirmed using blue native polyacrylamide gel electrophoresis with detergent-solubilized 293T cells expressing GFP-Tip^211-256^ or GFP-Tip CD71TM^211-256^. Truncated forms of the GFP fusion proteins, with short cytoplasmic domains, were used so as to minimize potential interactions with other cellular proteins, and to facilitate more accurate estimates of the size of the oligomeric protein. As shown in Figure 8B, a GFP fusion protein carrying the TM domain of wild type Tip (GFP-Tip^211-256^) migrated as a ∼150 kDa protein, whereas GFP-Tip CD71TM^211-256^ migrated as a ∼40 kD protein, corresponding to the size of the monomeric form of GFP-Tip CD71TM^211-256^. The size of the ∼150 kD protein complex is suggestive of homo-oligomers of four GFP-Tip^211-256^ monomers.

## Discussion

Lipid rafts contain proteins that retain their association with membrane lipids. These proteins are mostly GPI-anchored or acylated, but a few are transmembrane proteins, which are targeted to lipid rafts through their TM domain or through membrane-proximal determinants [21]. Here, we have found that the presence of a membrane-proximal amphipathic helix, located in Tip's cytoplasmic face, significantly contributed to Tip's localization in the lipid raft. Extensive mutagenesis analysis revealed that the residues forming both the hydrophobic ridge and the positively-charged face of the helical motif are important for Tip's efficient association with lipid rafts (Figure 2). The segregation of hydrophobic and polar residues into two opposite faces of the helical structure matches well with the chemistry of the membrane interface, and has been suggested to contribute to membrane adsorption [22],[23]. It has also been suggested that the amphipathic helical motif might target caveolin to lipid rafts through partial insertion of the hydrophobic ridge into lipid bilayer, and electrostatic interaction of the charged surface with phospholipids [24]. Another lipid raft-residing protein, α-synuclein [25], is also anchored to membranes by an elongated amphipathic helical structure [26]. Although the specificity of the amphipathic helical motifs for lipid rafts has been poorly defined, binding of α-synuclein to raft-like liposomes was shown to require acidic phospholipids, with a preference for phosphatidylserine [25]. In T cells, cholesterol and negatively-charged phospholipids are concentrated in the ordered raft domains upon antigenic stimulation [1]. Interestingly, a recent report showed that many signaling and transport proteins contain clusters of positively-charged amino acids, suggesting that those clusters could mediate the plasma membrane-targeting of proteins, through interaction with acidic phospholipids [27]. In this study, we showed that the amphipathic helical peptide of Tip specifically interacts with negatively-charged lipids such as PS, PA, PG, and PtdIns rather than neutral or amino phospholipids (Figure 6), suggesting that positively-charged amino acids within the amphipathic helical motif might associate with these negatively-charged lipids. This possibility is consistent with previous findings, which showed that the cytoplasmic leaflet of biological membranes is enriched in negatively-charged lipids, and that lipid rafts are enriched with PS, PA, and PG [28]. As suggested by the results of binding assays utilizing artificial liposomes containing cholesterol (Figure 6C), however, the presence of the hydrophobic ridge might restrict the interaction of the peptide with lipid rafts. In fact, α-synuclein binds more strongly to membranes containing low or no cholesterol [25] and the binding affinity of an amphipathic peptide to unilamellar vesicles is reduced by the presence of cholesterol [29]. The rigidifying effect of cholesterol on phospholipid acyl chains may limit the penetration of the peptide into the bilayer interior. Taken all together, the interaction of the amphipathic helical region of Tip with lipid bilayer might be heavily dependant on the membrane lipid composition.

Previously, we found that the TM domain of Tip is required for association with lipid rafts [11]. In this study, we found that the TM domain alone confers weak (∼20%) lipid raft association (Figure 1A) but is sufficient to mediate oligomerization of Tip (Figure 8). These results suggested that the TM domain might play a cooperative role in lipid raft association together with the lipid binding amphipathic helix; i.e. the both domains are required for the efficient association of Tip with lipid rafts. With regard to the relationship between lipid raft association and protein oligomerization, varying results have been reported. In some cases, self-assembling or ligand-induced oligomerization was required for proteins to associate efficiently with the lipid raft [30],[31], whereas oligomerization and lipid raft formation were independent in other cases [32]. Alanine scan mutagenesis was performed in an effort to elucidate the role of the Tip TM domain in lipid raft localization and oligomerization (Figure S8). A lipid raft fractionation assay using Tip mutants carrying four consecutive alanine residues in the TM domain showed that two mutants, carrying alanine residues in the regions 240–244 or 249–253, associate with lipid rafts to the same degree as wild type Tip, and that other mutants were able to associate with lipid rafts to a limited degree of approximately 40 to 47% (Figure S8B). These observations imply that the amino acids 231–241 and the TVLS motif, which are thought to interact with the inner and outer leaflets of biological membranes respectively, were both required for efficient lipid raft association, suggesting that the specific amino acid sequences comprising the Tip TM domain might contribute to the interaction with lipid raft domains. The immunoprecipitation assay, using cells expressing AU1-tagged wild type Tip and flag-tagged Tip mutants generated by the alanine scan mutagenesis, showed that all mutants tested co-precipitated with wild type Tip (Figure S8C). As such, oligomerization might be mediated by regions longer than four consecutive amino acids, or by multiple contacts within the TM domain, rather than by a single or local contact. Since no motif specific for the oligomerization of Tip could be identified, the precise relationship between protein oligomerization and lipid raft association has yet to be determined, and will be the topic of future studies.

It could be questioned whether the mutations in the amphipathic helix or transmembrane domain of Tip could affect the efficiency of membrane association itself rather than lipid raft localization. However, when we examined the membrane association of Tip and its mutants, Tip amp1 or Tip CD71TM, without detergent treatment during membrane fractionation, there was no significant difference in the degree of membrane association of the Tip proteins (Figure S9). Rather, the degree of membrane association of Tip amp1 or Tip CD71TM was slightly enhanced when compared to that of Tip wt. This result suggested that the mutations in the amphipathic helix or transmembrane domain could change the raft localization property of Tip without significantly affecting the efficiency of membrane association itself.

Vesicle trafficking involves dynamic remodeling of cellular membranes, for which the formation of local membrane curvature is a critical step [12],[33]. It has recently been shown that generation of membrane curvature can be driven by the interplay between lipids and proteins, through several mechanisms [12]. An emerging theme among these mechanisms is the involvement of amphipathic peptides that partially penetrate the lipid bilayer, acting as wedges. Active insertion of helical peptides into the bilayer results in an increase of surface area in one leaflet, possibly generating spontaneous curvature in the bilayer. This local curvature is subsequently sensed and stabilized by other domains of the curvature-forming proteins, or by coat proteins. For example, the N-terminal amphipathic helix found in the BAR domain of amphiphysin and endophilin has been shown to cause local membrane curvature, which is stabilized by a banana-shaped lipid-binding domain [19],[34],[35]. Helical domains found in other proteins such as epsin, Arf, and Sar1 were also shown to generate local membrane curvature in an induced manner and subsequently recruit coat proteins to stabilize the curvature [17],[18],[36]. The power of an amphipathic peptide to generate membrane deformation was previously demonstrated when a designed 18-mer peptide was shown to form extensive, 40–50 nm diameter tubules from liposomes [37]. They showed that that the deformation of liposomes depended on lipid composition and peptide properties such as length and the ratio of hydrophobic to hydrophilic amino acids. In the present study, we showed that an amphipathic 18-mer peptide derived from Tip's membrane-proximal helix can efficiently induce membrane deformation in an *in vitro* liposome tubulation assay (Figure 7). The point mutation of the conserved hydrophobic residues in this peptide into basic lysine residues improved liposome binding, in comparison to the wild type peptide, but abolished tubulation (Figure 7). These results suggest that Tip's membrane-proximal amphipathic helix is likely to alter membrane structure in a manner similar to that employed by other cellular proteins containing amphipathic helices. In fact, GFP fusion peptides encompassing the helical region and TM domain of Tip were detected in vesicular curvatures and multivesicular structures within endocytic vesicles of Jurkat T cells (Figure 5), whereas the mutant fusion peptides were not. Luminal budding of the limiting membrane and the formation of MVBs in the late endosomal pathway are efficient mechanisms for targeting membrane proteins/receptors to lysosomes for degradation [3]. Through its amphipathic helical motif, Tip might initiate the luminal budding step, which might be further enhanced by oligomerization through the TM domain. Considering that Tip's structural influence on the lipid bilayer is generated in the cytoplasmic face of endocytic vesicles, and that Tip lacks a curvature-sensing/stabilizing domain, the luminal budding would likely be assisted by other cellular proteins. Recently, it was shown that an inverse BAR domain-like mechanism in the proteins IRSp53 and MIM (missing-in-metastasis) induces a membrane curvature opposite to that of BAR domains, and deforms membranes by binding to their interior, resulting in plasma membrane protrusions rather than invaginations [38]. Thus, Tip-associated luminal budding may be facilitated by cellular proteins with an inverse BAR domain-like mechanism, which may be recruited through direct or indirect interactions with Tip. Vps (vaculolar protein sorting) proteins have been shown to be involved in lysosomal degradation of activated receptors through the MVB-sorting pathway in yeast and mammals [3],[39],[40]. Some mutations in those genes resulted in an enlarged late endosomal compartment, presumably because of an inability to invaginate the limiting membrane to form the MVB. It would be interesting to identify whether the protein sorting machineries could mediate inverse BAR domain-like functions in Tip associated-MVB formation.

Expression of Tip in T cells was previously reported to induce clustering of lipid raft domains as well as redistribution of TCR/CD3 complexes into lipid raft domains [9],[10]. Tip expression can also reorganize raft domains and enhance the recruitment of raft-resident components [11]. Similarly, T cell activation leads to the segregation of plasma membrane domains to form TCR signaling clusters, and this is accompanied by the condensation of the plasma membrane, driven by activation-induced protein-protein interactions such as anchorage to the cytoskeleton [41]. The clustered raft domain platforms are subsequently internalized and degraded in the lysosome to attenuate TCR signaling [42]. Previously, we showed that the TM domain is essential for the downregulation of TCR/CD3 complexes and CD4 by Tip [11]. Downregulation of the membrane proteins, however, is mediated through different mechanisms [9],[10]. Downregulation of TCR/CD3 complexes by Tip is dependent on its interaction with and kinase activity of Lck as well as the interaction with p80, whereas downregulation of CD4 by Tip is dependent on the physical association with Lck only. In the present study, we showed that Tip's membrane-proximal amphipathic helix, consisting of 14 amino acids, was essential for the selective downregulation of TCR/CD3 complexes but not for CD4. The effect of this short motif on the receptor trafficking might be linked to the functional properties of raft-targeting and membrane deformation, as mentioned above. Lysosomal targeting through MVB formation by the amphipathic helix and TM domain of Tip suggested how clustered TCR/CD3 complexes in lipid raft domains are targeted for lysosomal degradation in Tip-expressing T cells. In contrast to TCR/CD3 complexes, CD4 surface expression is downmodulated consistently by the Tip mutants lacking amphipathicity in their membrane proximal helix (Figure 3). As such, downregulation of CD4 surface expression could be mediated by different mechanisms. Although the molecular mechanisms of CD4 trafficking in resting and activated T cells are largely unknown, it is interesting to note that human immunodeficiency virus has dual arms, Nef and Vpu, to downregulate CD4 surface expression through distinct mechanisms [43]. Nef links mature CD4 to components of clathrin-dependent trafficking pathways at the plasma membrane, and perhaps also in intracellular compartments, leading to internalization and delivery of CD4 to lysosomes for degradation. Vpu, on the other hand, interacts with newly-synthesized CD4 in the endoplasmic reticulum, linking CD4 to the SCF ubiquitin ligase, and facilitating the entry of CD4 into the endoplasmic reticulum-associated degradation pathway. The Tip-associated molecular mechanisms controlling CD4 expression remain to be elucidated.

The phenotypic resemblance of Tip and TCR activation, leading to Lck activation, recruitment of TCRs to lipid rafts and finally to lysosomal degradation, suggests that HVS Tip may pirate cellular signaling molecules to emulate TCR stimulation for viral persistence and pathogenesis. Epstein-Barr virus LMP2A, a functional homologue of HVS Tip, has also been shown to be mimic the B cell receptor signal transduction to maintain viral latency, allowing long-term survival of infected B cell [44],[45]. This viral protein interacts with B-cell signaling proteins, such as Lyn and Syk, through its N-terminal cytoplasmic tail. LMP2A functions in lipid rafts to block translocation of the B-cell receptor into lipid rafts, which leads to inhibition of the subsequent signaling and accelerated internalization of the BCR-cell receptor upon stimulation. Thus, the study of HVS Tip may provide valuable insight into the conserved mechanisms employed by other γ-herpesvirus signal modulators to regulate lymphocyte functions and may have significant implications for the understanding of viral persistence and pathogenesis.

In summary, we have shown that a potential membrane-proximal amphipathic helix preceding the TM domain of Tip is essential for efficient lipid raft localization and selective downregulation of TCR/CD3, most likely through mechanisms involving membrane curvature and MVB formation in endocytic vesicles. Moreover, we could dissect the functional roles of the amphipathic helix and the TM domain in membrane deformation and oligomerization, respectively. These novel mechanisms of the viral protein could provide valuable insights into the functional relationship between lipid rafts and MVB formation and the molecular details of membrane trafficking of the key receptors in T cells.

## Materials and Methods

### Cell culture and reagents

Jurkat T cells were grown in RPMI, and 293T and HeLa cells were maintained in DME medium, supplemented with 10% FBS. Jurkat T cells were electroporated using a Bio-Rad electroporator at 260V and 975µF in serum-free RPMI medium. Lipofectamine2000 (Invitrogen) or calcium phosphate (Clontech) was used to induce transient expression of Tip in HeLa and 293T cells. Stable Jurkat T cell lines expressing Tip or its mutants were selected and maintained in the presence of puromycin (5 µg/ml). We tested the expression level of Tip or its mutants in each cell line by semi-quantitative RT-PCR using β-actin gene as internal control [46]and confirmed the similar level of expression in all the established cell lines (data not shown). An anti-GFP antibody (Santa Cruz Biotechnology), a CTB-HRP conjugate (Sigma), an anti-EEA1 antibody, and an anti-LAMP2 antibody (BD Bioscience) were used for immunoassays. DNA fragments encoding Tip and its mutants were cloned into pFJ, pBabe or p3xFlag_CMV vectors (Sigma) using methods described previously [11]. GFP fusion proteins containing Tip or its mutants were made using pEGFP-C2 plasmids (Clontech). PCR-based mutagenesis was performed to create the Tip mutants, using sequences described previously [10],[11]. A peptide corresponding to the amphipathic helical region of Tip (Tip wt^211-228^, ANERNIVKDLKRLENKIN) and a mutant peptide in which hydrophobic residues were replaced with lysines (underlined; Tip amp1^211-228^, ANERNKVKDKKRKENKKN) were synthesized by Peptron Inc. A lysine residue was added to the C-terminus of each peptide to allow them to be conjugated with biotin.

### Isolation of lipid rafts

Lipid rafts were isolated using a method involving flotation on discontinuous sucrose gradients [10]. Briefly, 5×10^7^ 293T cells were washed with ice-cold PBS and lysed for 30 min on ice in 1% Triton X-100 in TNEV buffer (10 mM Tris-HCl, pH 7.5, 150 mM NaCl, 5 mM EDTA) containing phosphatase inhibitors and protease inhibitor cocktail (Roche). The lysates were further homogenized in a Wheaton loose-fitting Dounce homogenizer. Nuclei and cellular debris were pelleted by centrifugation at 900×*g* for 10 min. For the discontinuous sucrose gradient, 0.5 ml of cleared cell lysates were mixed with 0.5 ml of 85% sucrose in TNEV and transferred to a Beckman 14×89 mm centrifuge tube. Diluted lysates were overlaid with 4 ml of 35% sucrose in TNEV and finally 1 ml 5% sucrose in TNEV. Samples were then centrifuged in an SW41 rotor at 200,000×*g* for 20 h at 4°C, and 0.5 ml fractions were collected from the top of the gradient.

### Preparation of the membrane-enriched fraction

Membrane-enriched fraction was prepared to examine the efficiency of membrane association of Tip and its mutants as described elsewhere with slight modification [47]. In brief, 293 T cells expressing GFP fusion proteins containing Tip or its mutants were harvested and resuspended in lysis buffer (50 mM Tris, pH 7.8, 250 mM Sucrose, and 2 mM EDTA) with protease inhibitor cocktail (Roche). After incubation on ice for 10 min, cells were lysed by 30 strokes using Dounce homogenizer at 4°C. Cellular debris and nuclei were removed by centrifugation at 1000×g for 10 min at 4°C. The postnuclear supernatant was layered onto a 60% sucrose cushion and centrifuged at 160,000×g for 1 h at 4°C. The membrane fraction on top of the sucrose cushion was collected, diluted 1∶2 with cold phosphate-buffered saline (PBS; 100 mM phosphate, 150 mM NaCl, pH 7.2) and pelleted at 100,000×g for 1 h at 4°C. The supernatant was discarded and the membrane pellet was rinsed twice with cold PBS, and pelleted at 20,000×g for 30 min at 4°C. The enriched membrane fraction was further used for SDS-PAGE and subsequent immunoblot assay.

### Blue Native PAGE

Blue Native PAGE was performed as described previously [48] with slight modifications. Homogenized cells were solubilized by adding Triton X-100 to a final concentration of 2.5%. After removing cellular debris by centrifugation, the whole-cell lysates were collected and resolved by native gel (10%) electrophoresis. Resolved proteins were transferred to a PVDF membrane and detected by immunoblot assay. Aldorase from rabbit muscle (∼160 kDa, Sigma) and bovine serum albumin (monomer: ∼66 kDa, dimer ∼132 kDa, Sigma) were used as molecular weight markers.

### Flow cytometry

Cells (5×10^5^) were washed with RPMI medium containing 10% fetal calf serum, and incubated with fluorescein isothiocyanate-conjugated or phycoerythrin-conjugated monoclonal antibodies for 30 min at 4°C. After washing, each sample was fixed with 4% paraformaldehyde solution and flow cytometric analysis was performed with a FACScan (Becton Dickinson Co.). Antibodies against CD3 (SK7), CD4 (Leu-3a), CD45 (HI30), and αβTCR were purchased from BD Pharmingen.

### Confocal immunofluorescence

Cells were fixed with 4% paraformaldehyde for 15 min, permeabilized with 0.2% Triton X-100 for 15 min, and reacted with primary antibodies in PBS for 30 min at room temperature. Alexa 488- or Alexa 594-conjugated anti-rabbit or anti-mouse antibodies (Molecular Probes) were used as secondary antibodies. Confocal microscopy was performed using an Olympus FV1000 laser-scanning microscope (Olympus) fitted with a 60× Olympus objective. Images were collected at 512×512 pixel resolution using Olympus imaging software. The stained cells were optically sectioned in the z-axis, and the images in the different channels (photo multiplier tubes) were collected sequentially. The images were rendered using Olympus Fluoview v1.6b or Adobe Photoshop software. To quantify the degree of relative colocalization, we obtained the Pearson correlation coefficient (R) values, which are standard measures of colocalization [49]. The R values were calculated using the Olympus Fluoview v1.6b colocalization module which generates a “colocalized” image from two channels.

### Immunoprecipitation and immunoblot

For immunoprecipitation, cells were harvested and resuspended in lysis buffer (150 mM NaCl, 0.5% Nonidet P-40, and 50 mM HEPES buffer, pH 7.4) containing protease inhibitors. Immunoprecipitated proteins from precleared cell lysates were used for immunoblot. For immunoblot, polypeptides were resolved by SDS-PAGE and transferred to a PVDF membrane. Immunoblot detection was performed with a 1∶1000 or 1∶3000 dilution of primary antibody and an enhanced chemiluminescence system (Pierce).

### Peptide-lipid or -liposome binding assay

Membrane lipid strips and arrays (Echelon Biosciences) were used for peptide-lipid binding assays according to the manufacturer's instructions. Peptides (0.4 µM) were incubated overnight at 4°C and detected with streptavidin-HRP conjugates. Densitometric analysis was applied to determine the relative affinity of peptide binding to the various lipids. After subtracting background values, numerical densitometric values were attributed to each of the five concentrations measured. The highest value, for binding of peptides to 100 pmol of lipids, was arbitrarily assigned “100% binding” and all other lipids were normalized in comparison to that maximum binding value. Synthetic liposomes were made using phosphatidylcholine (65% mol/mol), phosphatidylserine (25% mol/mol), and cholesterol (10% mol/mol; Avanti Polar Lipids Inc.), as described previously [19]. To achieve desired diameters, the liposomes were extruded more than 10 times through a polycarbonate membrane (Avanti). The size of the liposomes was measured by laser light scattering analysis (Brookhaven Instruments Co.). For the peptide binding assays, liposomes were diluted in 100 µl of binding buffer (20 mM HEPES, pH 7.4, 150 mM NaCl) at a final lipid concentration of 2 mM and incubated for 10 min at room temperature with peptides (4 µM). Liposome-protein complexes were recovered by centrifugation (100,000×*g*) at room temperature for 20 min, the supernatant was completely removed, and sedimented liposomes were solubilized in SDS sample buffer. The peptides in the supernatant and pellet were subjected to SDS-PAGE using 16% tricine gels, and analyzed as described above.

### Electron microscopy

Jurkat T cells expressing GFP fusion proteins were fixed in 0.5% glutaraldehyde and 4% paraformaldehyde in 0.05 M sodium cacodylate buffer (pH 7.2) at 4°C for 2 h. Ultrathin sections (50 nm in thickness) were cut using an ultramicrotome (MT-X; RMC) and stained with an anti-GFP primary antibody and an anti-rabbit IgG secondary antibody conjugated with 10 nm gold particles (Sigma). Sections were then stained with 2% uranyl acetate and Reynolds' lead citrate, and examined by transmission electron microscopy (LIBRA 120; Carl Zeiss) at an accelerating voltage of 120 kV. Negative control experiments were also performed to ensure the specificity of the labeling by replacing the primary antibody with rabbit preimmune serum. Liposomes incubated with peptides as described in liposome binding assays were adsorbed onto carbon-coated copper grids, stained with uranyl acetate, and then observed by electron microscopy.

### Accession numbers

The GenBank (http://www.ncbi.nlm.nih.gov/Genbank) accession numbers for Tip and Tio proteins used in this paper are Tip C488 (AAA72928), Tip C484 (P88825), Tip C484-77 (P25049), and Tio (AAC95538).

## Supporting Information

Figure S1 Schematic representation of GFP fusion proteins of wild type or mutant Tip. In GFP-Tip amp2, which is not shown here, the positively-charged amino acids of the amphipathic helix are point-mutated into alanine (Figure 2A).
(0.05 MB PDF)

Figure S2 Lipid raft association of flag-tagged Tip or its deletion mutant. 293T cells were transfected with plasmids encoding Tip or its deletion mutants as flag-tagged proteins, and processed for lipid raft fractionation. Proteins from each fraction of the sucrose gradient were subjected to immunoblotting with an anti-flag antibody to detect Tip or its mutants. CTB-HRP was used to confirm the localization and integrity of the lipid rafts. The degree of lipid raft association was estimated by densitometry analysis.
(0.08 MB PDF)

Figure S3 Secondary structure prediction of the N-terminal region of Tip from Herpevirus saimiri strains and Tio from Herpesvirus ateles, performed using PSIPRED (http://bioinf.cs.ucl.ac.uk/psipred/psiform.html). The predicted amphipathic helix (red box) and the transmembrane domain (blue box) are indicated.
(0.14 MB PDF)

Figure S4 Quantification of colocalization of Tip wt and Tip amp1 with early endosomes, late endosome/lysosomes, or CD3ζ. Jurkat T cells electroporated with plasmids encoding Tip or Tip amp1 were analyzed for Tip, Tip amp1, EEA1, LAMP2, CD3ζ colocalization as described in Fig. 3B. Pearson coefficient (R) values were obtained from 10 to 20 cells as described in materials and methods.
(0.05 MB PDF)

Figure S5 Quantification of colocalization of Tip wt211-256, Tip amp1211-256, and Tip CD71TM211-256 with early endosomes or late endosome/lysosomes. Jurkat T cells expressing the GFP-fusion proteins were analyzed for colocalization with EEA1 or LAMP2 as described in Fig. 4A (A). In addition, HeLa cells expressing the GFP-fusion proteins were also analyzed for colocalization with LAMP2 as described in Fig. 4B (B). Pearson coefficient (R) values were obtained from 10 to 20 cells as described in materials and methods.
(0.05 MB PDF)

Figure S6 Quantitative analysis of binding between peptides and lipids. Numerical densitometric values obtained from Figure 5B were plotted as percentages of the maximum binding of peptides to each lipids (the highest value, for peptide binding to 100 pmol of lipids, was arbitrarily assigned “100% binding”). To estimate the saturated binding, 3-parameter sigmoidal regressions were performed (dotted line).
(0.05 MB PDF)

Figure S7 Size changes of liposomes following addition of amphipathic peptides. For the quantitative analysis of liposome tubulation, a laser light scattering assay was used, in which the morphological changes are estimated indirectly by measuring the size changes of liposomes. The liposomal size distribution was significantly altered by the addition of the amphipathic helical peptide (Tip wt211-228), in comparison to the distribution of the control liposomes (absence of peptide) or that of liposomes incubated with mutant peptide (Tip amp1211-228).
(0.01 MB PDF)

Figure S8 TM domain amino acids contributing to lipid raft association, identified by alanine scan mutagenesis. (A) Sequences of wild type Tip, and its mutants carrying alanine scan mutations in their TM domain. (B) 293T cells transiently expressing wild type Tip or its mutants were lysed and processed for lipid raft fractionation. Proteins from each fraction of the sucrose gradient were subjected to immunoblotting with an anti-Flag antibody to detect Tip or its mutants. CTB-HRP was used to confirm the localization and integrity of lipid rafts. The degree of lipid raft association was estimated by densitometry analysis and indicated as a percentage, as shown on the right side of each panel. (C) 293T cells were transfected with plasmids encoding AU1-tagged wild type Tip and Flag-tagged wild type Tip or its mutants. Cell lysates were analyzed by immunoprecipitation with an anti-Flag antibody followed by SDS-PAGE and immunoblotting with an anti-AU1 antibody or an anti-Flag antibody.
(0.20 MB PDF)

Figure S9 The effect of mutations in amphipathic helix or transmembrane domain of Tip on the membrane association. The efficiency of membrane association of Tip and its mutants were measured by comparing the ratio of the proteins in whole cell lysate (WCL) and membrane-enriched fraction (MF). GFP fusion proteins in the 2% of WCL or 10% of MF were detected by immunoblot and the ratio of protein level in each sample was measured by densitometric analysis.
(0.06 MB PDF)
