# Supplementary material for: Role of Amphipathic Helix of a Herpesviral Protein in Membrane Deformation and T Cell Receptor Downregulation
Source: PLoS Pathog. 2008 Nov 21;4(11):e1000209. doi: 10.1371/journal.ppat.1000209 (PMC2581436; doi:10.1371/journal.ppat.1000209)

Figure S1. Schematic representation of GFP fusion proteins of wild type or mutant Tip. In GFP-Tip amp2, which is not shown here, the positively-charged amino acids of the amphipathic helix are point-mutated into alanine (Figure 2A).

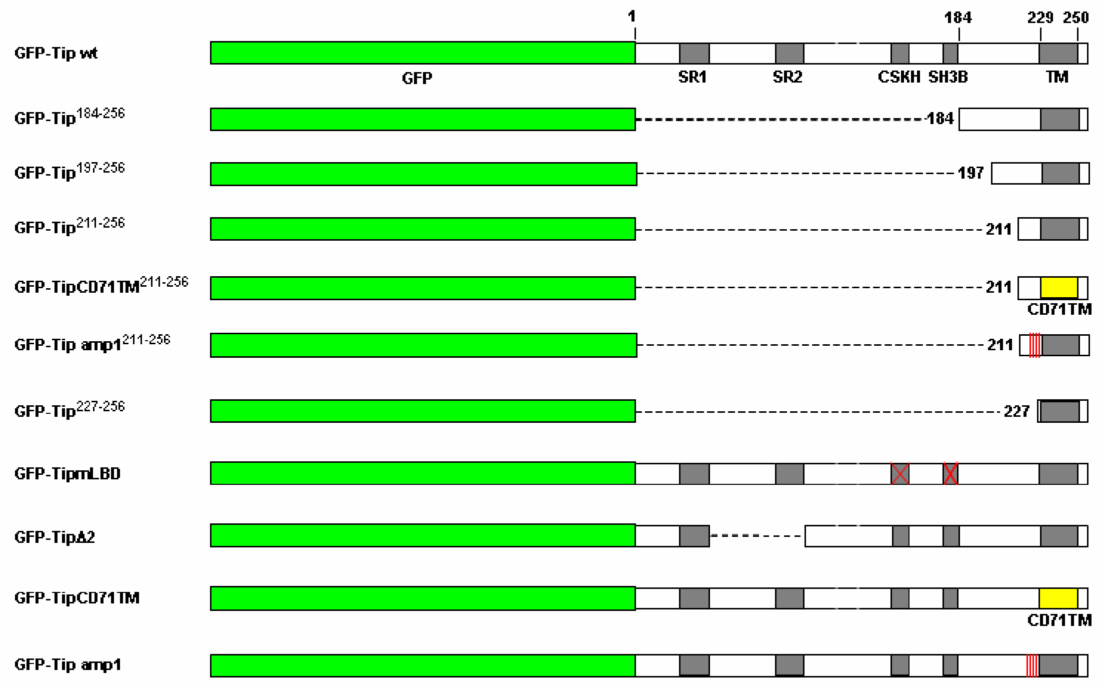

Supplement: Figure S1 — Schematic representation of GFP fusion proteins of wild type or mutant Tip. In GFP-Tip amp2, which is not shown here, the positively-charged amino acids of the amphipathic helix are point-mutated into alanine (Figure 2A). (0.05 MB PDF) [file ppat.1000209.s001.pdf]
