# Supplementary material for: Role of Amphipathic Helix of a Herpesviral Protein in Membrane Deformation and T Cell Receptor Downregulation
Source: PLoS Pathog. 2008 Nov 21;4(11):e1000209. doi: 10.1371/journal.ppat.1000209 (PMC2581436; doi:10.1371/journal.ppat.1000209)

Figure S2. Lipid raft association of flag-tagged Tip or its deletion mutants. 293T cells were transfected with plasmids encoding Tip or its deletion mutants as flag-tagged proteins, and processed for lipid raft fractionation. Proteins from each fraction of the sucrose gradient were subjected to immunoblotting with an anti-flag antibody to detect Tip or its mutants. CTB-HRP was used to confirm the localization and integrity of the lipid rafts. The degree of lipid raft association was estimated by densitometry analysis.

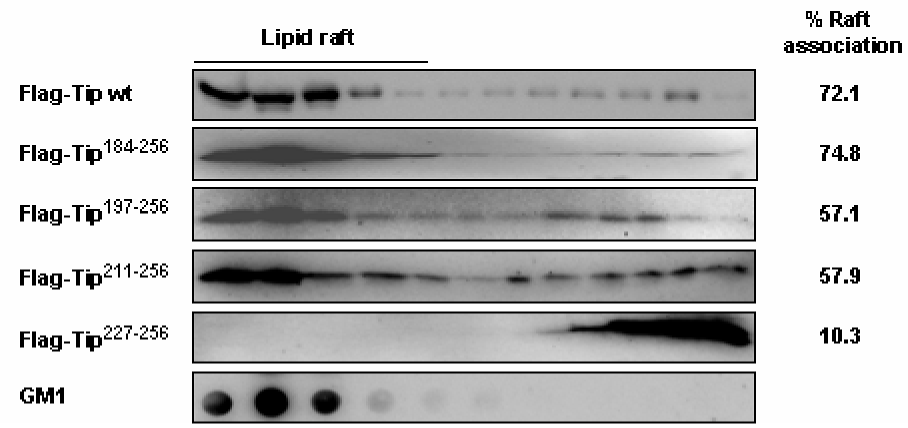

Supplement: Figure S2 — Lipid raft association of flag-tagged Tip or its deletion mutant. 293T cells were transfected with plasmids encoding Tip or its deletion mutants as flag-tagged proteins, and processed for lipid raft fractionation. Proteins from each fraction of the sucrose gradient were subjected to immunoblotting with an anti-flag antibody to detect Tip or its mutants. CTB-HRP was used to confirm the localization and integrity of the lipid rafts. The degree of lipid raft association was estimated by densitometry analysis. (0.08 MB PDF) [file ppat.1000209.s002.pdf]
