# Supplementary material for: Role of Amphipathic Helix of a Herpesviral Protein in Membrane Deformation and T Cell Receptor Downregulation
Source: PLoS Pathog. 2008 Nov 21;4(11):e1000209. doi: 10.1371/journal.ppat.1000209 (PMC2581436; doi:10.1371/journal.ppat.1000209)

Figure S4. Quantification of colocalization of Tip wt and Tip amp1 with early endosomes, late endosome/lysosomes, or CD3ζ. Jurkat T cells electroporated with plasmids encoding Tip or Tip amp1 were analyzed for Tip, Tip amp1, EEA1, LAMP2, CD3ζ colocalization as described in Fig. 3B. Pearson coefficient (R) values were obtained from 10 to 20 cells as described in materials and methods.

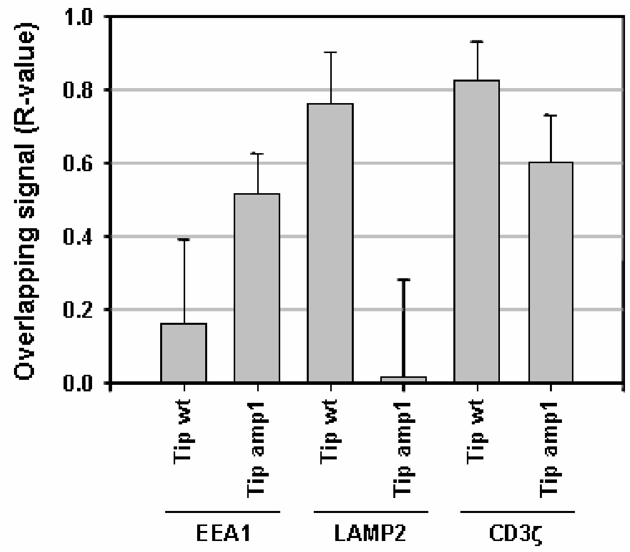

Supplement: Figure S4 — Quantification of colocalization of Tip wt and Tip amp1 with early endosomes, late endosome/lysosomes, or CD3ζ. Jurkat T cells electroporated with plasmids encoding Tip or Tip amp1 were analyzed for Tip, Tip amp1, EEA1, LAMP2, CD3ζ colocalization as described in Fig. 3B. Pearson coefficient (R) values were obtained from 10 to 20 cells as described in materials and methods. (0.05 MB PDF) [file ppat.1000209.s004.pdf]
