# Supplementary material for: Role of Amphipathic Helix of a Herpesviral Protein in Membrane Deformation and T Cell Receptor Downregulation
Source: PLoS Pathog. 2008 Nov 21;4(11):e1000209. doi: 10.1371/journal.ppat.1000209 (PMC2581436; doi:10.1371/journal.ppat.1000209)

Figure S5. Quantification of colocalization of Tip wt$^{211-256}$, Tip amp1$^{211-256}$, and Tip CD71TM$^{211-256}$ with early endosomes or late endosome/lysosomes. Jurkat T cells expressing the GFP-fusion proteins were analyzed for colocalization with EEA1 or LAMP2 as described in Fig. 4A, (A). In addition, HeLa cells expressing the GFP-fusion proteins were also analyzed for colocalization with LAMP2 as described in Fig. 4B (B). Pearson coefficient (R) values were obtained from 10 to 20 cells as described in materials and methods.

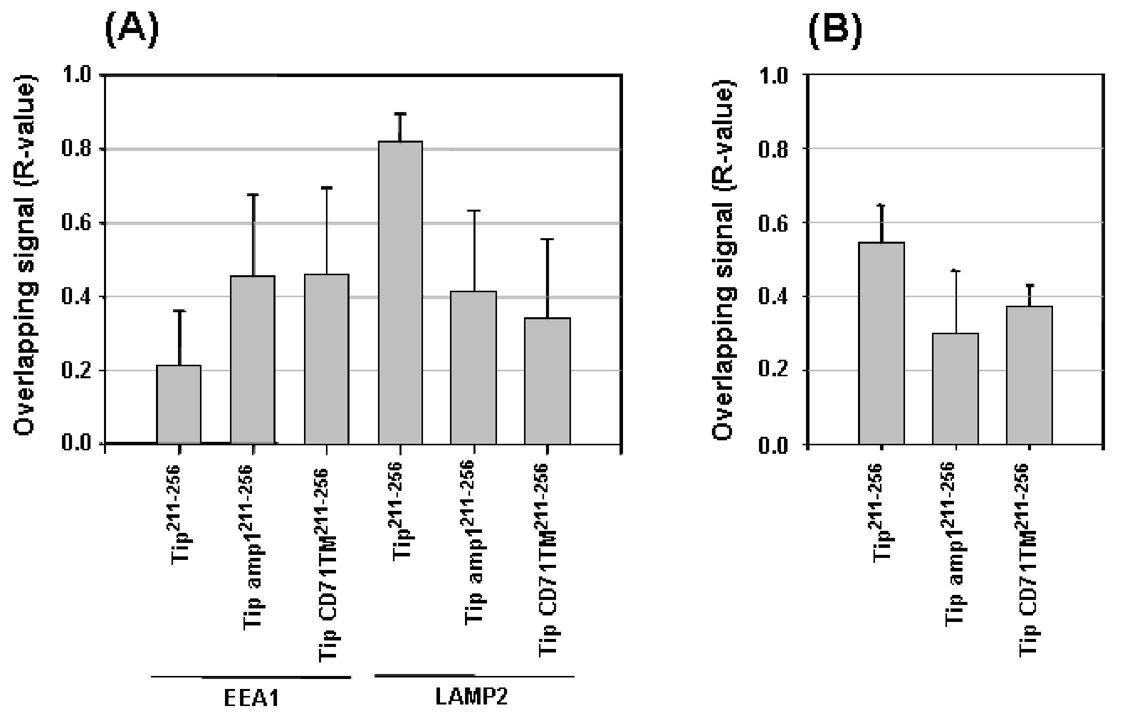

Supplement: Figure S5 — Quantification of colocalization of Tip wt211-256, Tip amp1211-256, and Tip CD71TM211-256 with early endosomes or late endosome/lysosomes. Jurkat T cells expressing the GFP-fusion proteins were analyzed for colocalization with EEA1 or LAMP2 as described in Fig. 4A (A). In addition, HeLa cells expressing the GFP-fusion proteins were also analyzed for colocalization with LAMP2 as described in Fig. 4B (B). Pearson coefficient (R) values were obtained from 10 to 20 cells as described in materials and methods. (0.05 MB PDF) [file ppat.1000209.s005.pdf]
