# Supplementary material for: Role of Amphipathic Helix of a Herpesviral Protein in Membrane Deformation and T Cell Receptor Downregulation
Source: PLoS Pathog. 2008 Nov 21;4(11):e1000209. doi: 10.1371/journal.ppat.1000209 (PMC2581436; doi:10.1371/journal.ppat.1000209)

Figure S6. Quantitative analysis of binding between peptides and lipids. Numerical densitometric values obtained from Figure 5B were plotted as percentages of the maximum binding of peptides to each lipids (the highest value, for peptide binding to 100 pmol of lipids, was arbitrarily assigned "100% binding"). To estimate the saturated binding, 3-parameter sigmoidal regressions were performed (dotted line).

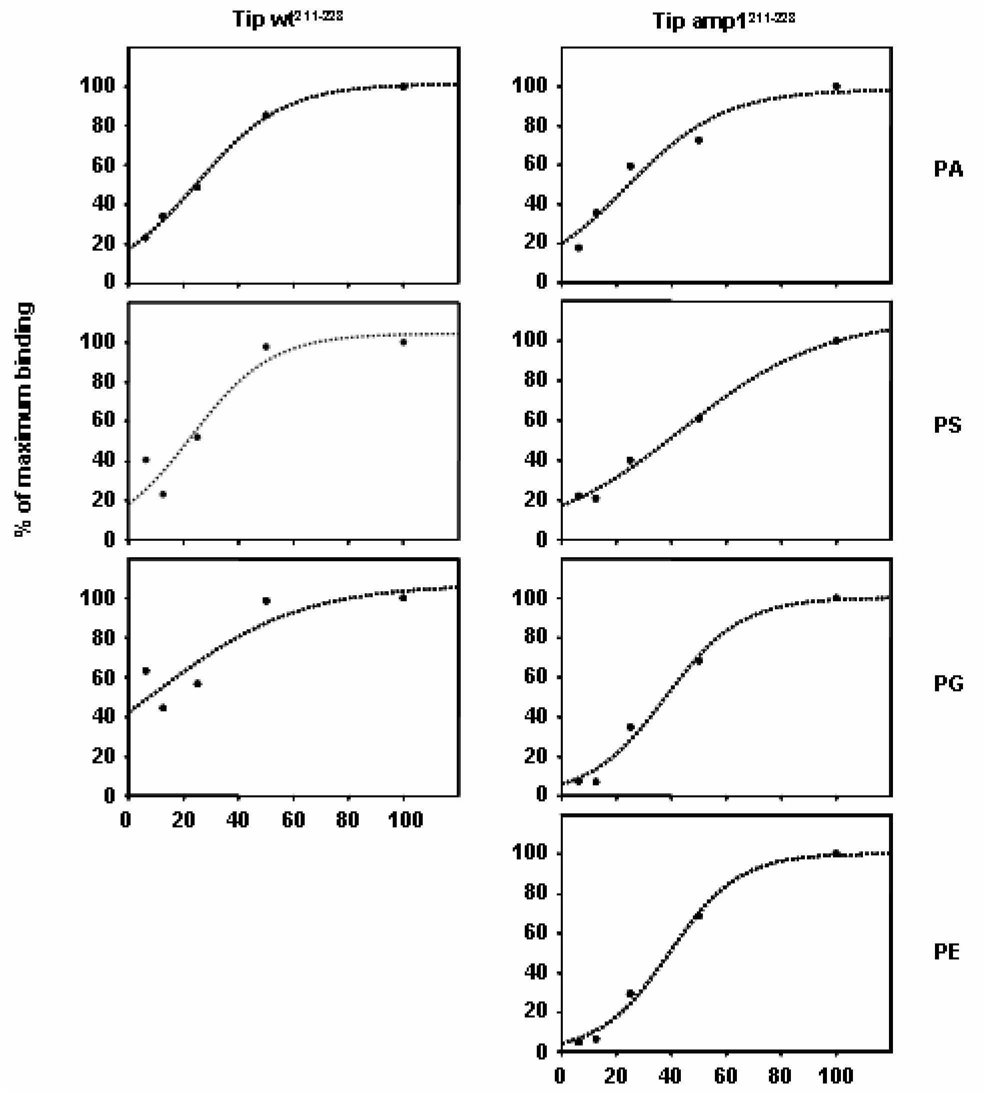

Supplement: Figure S6 — Quantitative analysis of binding between peptides and lipids. Numerical densitometric values obtained from Figure 5B were plotted as percentages of the maximum binding of peptides to each lipids (the highest value, for peptide binding to 100 pmol of lipids, was arbitrarily assigned “100% binding”). To estimate the saturated binding, 3-parameter sigmoidal regressions were performed (dotted line). (0.05 MB PDF) [file ppat.1000209.s006.pdf]
