# Supplementary material for: Role of Amphipathic Helix of a Herpesviral Protein in Membrane Deformation and T Cell Receptor Downregulation
Source: PLoS Pathog. 2008 Nov 21;4(11):e1000209. doi: 10.1371/journal.ppat.1000209 (PMC2581436; doi:10.1371/journal.ppat.1000209)

Figure S7. Size changes of liposomes following addition of amphipathic peptides. For the quantitative analysis of liposome tubulation, a laser light scattering assay was used, in which the morphological changes are estimated indirectly by measuring the size changes of liposomes. The liposomal size distribution was significantly altered by the addition of the amphipathic helical peptide (Tip wt$^{211\text{-}228}$), in comparison to the distribution of the control liposomes (absence of peptide) or that of liposomes incubated with mutant peptide (Tip amp1$^{211\text{-}228}$).

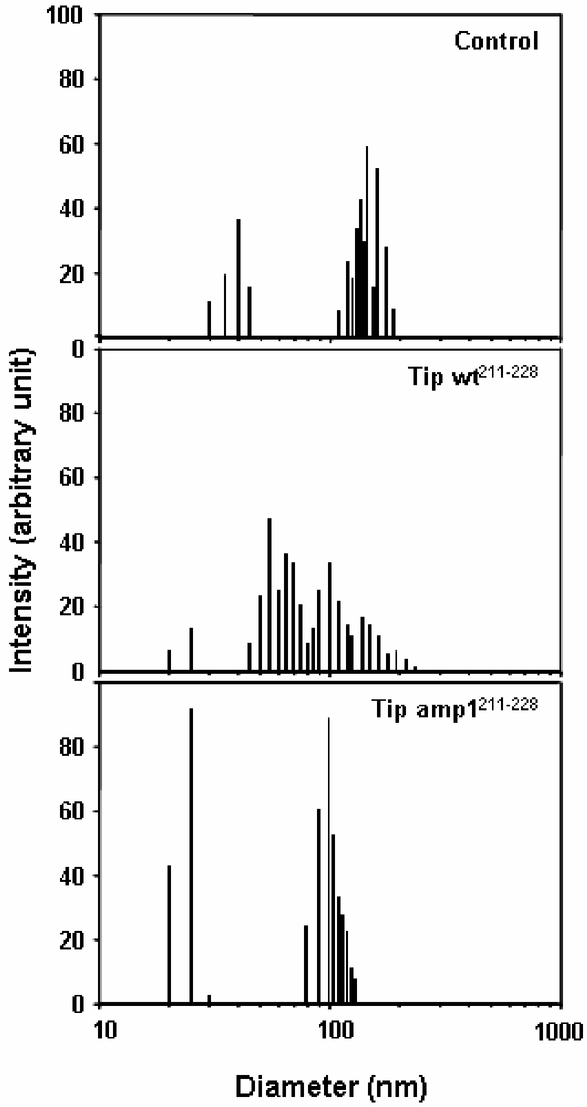

Supplement: Figure S7 — Size changes of liposomes following addition of amphipathic peptides. For the quantitative analysis of liposome tubulation, a laser light scattering assay was used, in which the morphological changes are estimated indirectly by measuring the size changes of liposomes. The liposomal size distribution was significantly altered by the addition of the amphipathic helical peptide (Tip wt211-228), in comparison to the distribution of the control liposomes (absence of peptide) or that of liposomes incubated with mutant peptide (Tip amp1211-228). (0.01 MB PDF) [file ppat.1000209.s007.pdf]
