# Supplementary material for: Role of Amphipathic Helix of a Herpesviral Protein in Membrane Deformation and T Cell Receptor Downregulation
Source: PLoS Pathog. 2008 Nov 21;4(11):e1000209. doi: 10.1371/journal.ppat.1000209 (PMC2581436; doi:10.1371/journal.ppat.1000209)
Supplement: Figure S8 — TM domain amino acids contributing to lipid raft association, identified by alanine scan mutagenesis. (A) Sequences of wild type Tip, and its mutants carrying alanine scan mutations in their TM domain. (B) 293T cells transiently expressing wild type Tip or its mutants were lysed and processed for lipid raft fractionation. Proteins from each fraction of the sucrose gradient were subjected to immunoblotting with an anti-Flag antibody to detect Tip or its mutants. CTB-HRP was used to confirm the localization and integrity of lipid rafts. The degree of lipid raft association was estimated by densitometry analysis and indicated as a percentage, as shown on the right side of each panel. (C) 293T cells were transfected with plasmids encoding AU1-tagged wild type Tip and Flag-tagged wild type Tip or its mutants. Cell lysates were analyzed by immunoprecipitation with an anti-Flag antibody followed by SDS-PAGE and immunoblotting with an anti-AU1 antibody or an anti-Flag antibody. (0.20 MB PDF) [file ppat.1000209.s008.pdf]

Figure S8. TM domain amino acids contributing to lipid raft association, identified by alanine scan mutagenesis. (A) Sequences of wild type Tip, and its mutants carrying alanine scan mutations in their TM domain. (B) 293T cells transiently expressing wild type Tip or its mutants were lysed and processed for lipid raft fractionation. Proteins from each fraction of the sucrose gradient were subjected to immunoblotting with an anti-Flag antibody to detect Tip or its mutants. CTB-HRP was used to confirm the localization and integrity of lipid rafts. The degree of lipid raft association was estimated by densitometry analysis and indicated as a percentage, as shown on the right side of each panel. (C) 293T cells were transfected with plasmids encoding AU1-tagged wild type Tip and Flag-tagged wild type Tip or its mutants. Cell lysates were analyzed by immunoprecipitation with an anti-Flag antibody followed by SDS-PAGE and immunoblotting with an anti-AU1 antibody or an anti-Flag antibody.

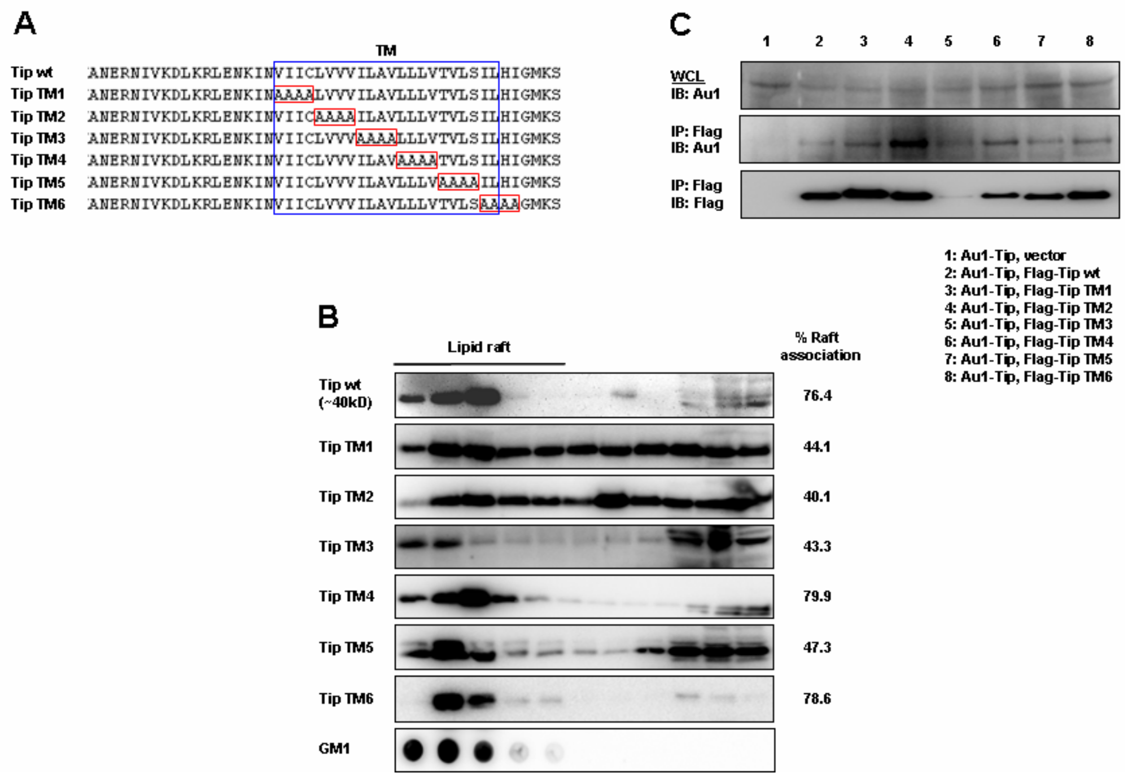
A
TM
Tip wt ANERNIVKDLKRLENKINVIICLVVVILAVLLLVTVLSILHIGMKS
Tip TM1 ANERNIVKDLKRLENKINAAAALVVVILAVLLLVTVLSILHIGMKS
Tip TM2 ANERNIVKDLKRLENKINVIICAAAAILAVLLLVTVLSILHIGMKS
Tip TM3 ANERNIVKDLKRLENKINVIICLVVVAAAALLLVTVLSILHIGMKS
Tip TM4 ANERNIVKDLKRLENKINVIICLVVVILAVAAAATVLSILHIGMKS
Tip TM5 ANERNIVKDLKRLENKINVIICLVVVILAVLLLVAAAAILHIGMKS
Tip TM6 ANERNIVKDLKRLENKINVIICLVVVILAVLLLVTVLSAAAAGMKS
C
1 2 3 4 5 6 7 8
WCL
IB: Au1
IP: Flag
IB: Au1
IP: Flag
IB: Flag
1: Au1-Tip, vector
2: Au1-Tip, Flag-Tip wt
3: Au1-Tip, Flag-Tip TM1
4: Au1-Tip, Flag-Tip TM2
5: Au1-Tip, Flag-Tip TM3
6: Au1-Tip, Flag-Tip TM4
7: Au1-Tip, Flag-Tip TM5
8: Au1-Tip, Flag-Tip TM6
B
Lipid raft
% Raft association
Tip wt (~40kD) 76.4
Tip TM1 44.1
Tip TM2 40.1
Tip TM3 43.3
Tip TM4 79.9
Tip TM5 47.3
Tip TM6 78.6
GM1
