# Supplementary material for: Role of Amphipathic Helix of a Herpesviral Protein in Membrane Deformation and T Cell Receptor Downregulation
Source: PLoS Pathog. 2008 Nov 21;4(11):e1000209. doi: 10.1371/journal.ppat.1000209 (PMC2581436; doi:10.1371/journal.ppat.1000209)

Figure S9. The effect of mutations in amphipathic helix or transmembrane domain of Tip on the membrane association. The efficiency of membrane association of Tip and its mutants were measured by comparing the ratio of the proteins in whole cell lysate (WCL) and membrane-enriched fraction (MF). GFP fusion proteins in the 2% of WCL or 10% of MF were detected by immunoblot and the ratio of protein level in each sample was measured by densitometric analysis.

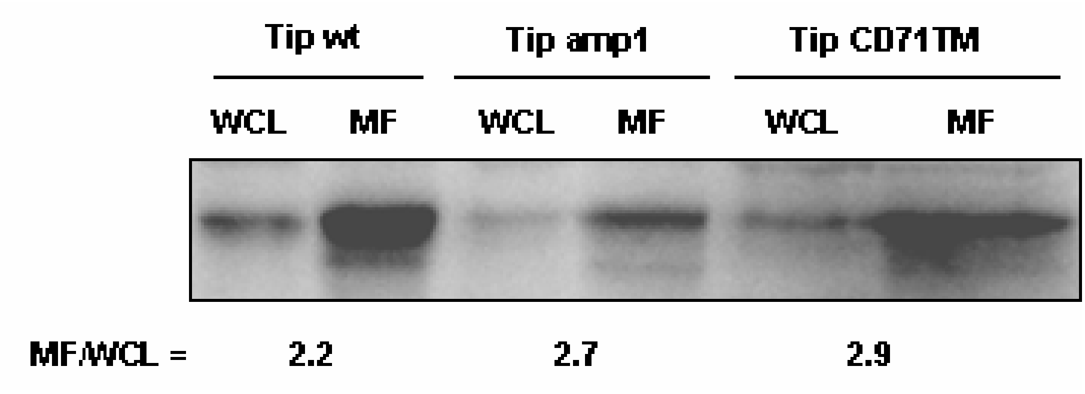

Supplement: Figure S9 — The effect of mutations in amphipathic helix or transmembrane domain of Tip on the membrane association. The efficiency of membrane association of Tip and its mutants were measured by comparing the ratio of the proteins in whole cell lysate (WCL) and membrane-enriched fraction (MF). GFP fusion proteins in the 2% of WCL or 10% of MF were detected by immunoblot and the ratio of protein level in each sample was measured by densitometric analysis. (0.06 MB PDF) [file ppat.1000209.s009.pdf]
