# Supplementary material for: Role of Amphipathic Helix of a Herpesviral Protein in Membrane Deformation and T Cell Receptor Downregulation
Source: PLoS Pathog. 2008 Nov 21;4(11):e1000209. doi: 10.1371/journal.ppat.1000209 (PMC2581436; doi:10.1371/journal.ppat.1000209)
Supplement: Figure S3 — Secondary structure prediction of the N-terminal region of Tip from Herpevirus saimiri strains and Tio from Herpesvirus ateles, performed using PSIPRED (http://bioinf.cs.ucl.ac.uk/psipred/psiform.html). The predicted amphipathic helix (red box) and the transmembrane domain (blue box) are indicated. (0.14 MB PDF) [file ppat.1000209.s003.pdf]

Figure S3. Secondary structure prediction of the N-terminal region of Tip from Herpevirus saimiri strains and Tio from Herpesvirus ateles, performed using PSIPRED (http://bioinf.cs.ucl.ac.uk/psipred/psiform.html). The predicted amphipathic helix (red box) and the transmembrane domain (blue box) are indicated.

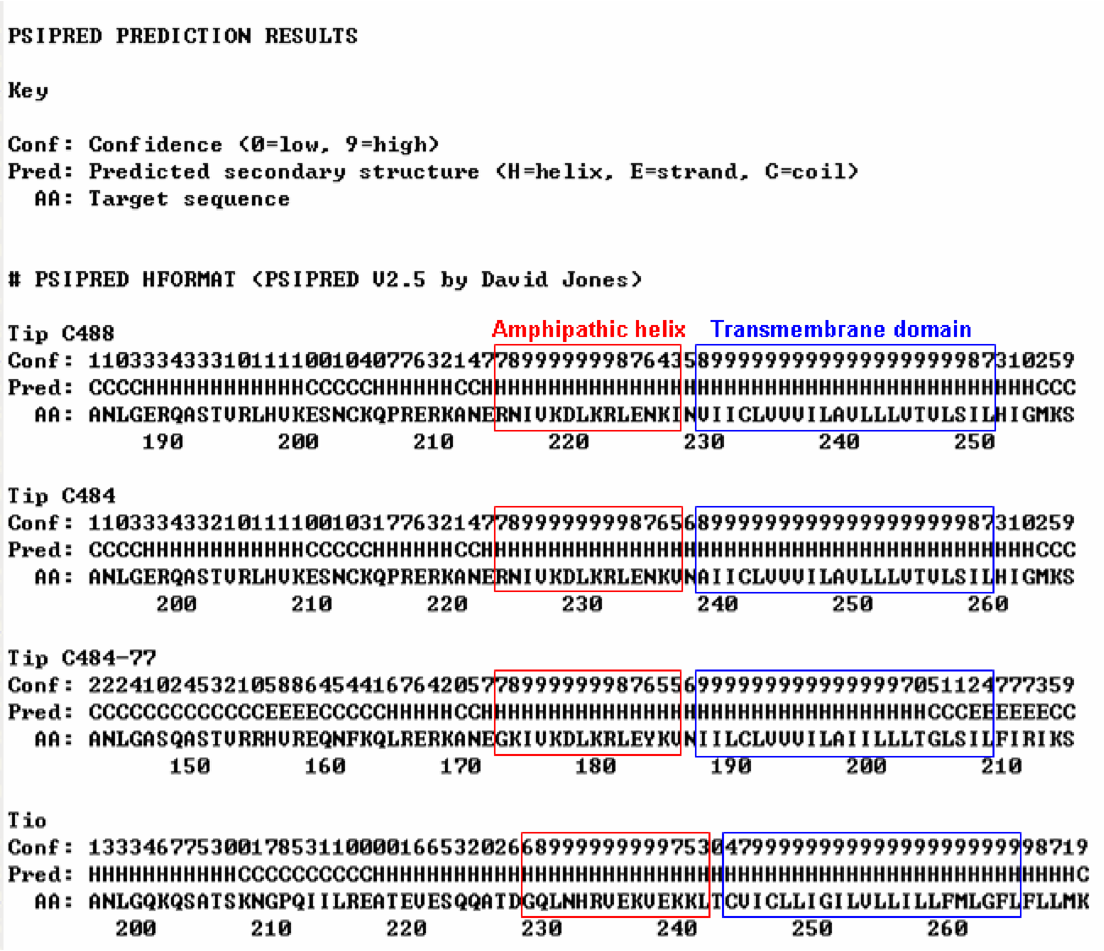

```
PSIPRED PREDICTION RESULTS

Key

Conf: Confidence (0=low, 9=high)
Pred: Predicted secondary structure (H=helix, E=strand, C=coil)
  AA: Target sequence


# PSIPRED HFORMAT (PSIPRED V2.5 by David Jones)

Tip C488                                           Amphipathic helix   Transmembrane domain
Conf: 1103334333101111001040776321477899999999987643589999999999999999999998731 0259
Pred: CCCCHHHHHHHHHHHHHCCCCCHHHHHHCCHHHHHHHHHHHHHHHHHHHHHHHHHHHHHHHHHHHHHHHHHHHHHHCCC
  AA: ANLGERQASTVRLHVKESNCKQPRERKANERNIVKDLKRLENKINVIICLVVVILAVLLLVTVLSILHIGMKS
             190       200       210       220       230       240       250

Tip C484
Conf: 11033343321011110010317763214778999999999876568999999999999999999999873 10259
Pred: CCCCHHHHHHHHHHHHHCCCCCHHHHHHCCHHHHHHHHHHHHHHHHHHHHHHHHHHHHHHHHHHHHHHHHHHHHHHCCC
  AA: ANLGERQASTVRLHVKESNCKQPRERKANERNIVKDLKRLENKVNAIICLVVVILAVLLLVTVLSILHIGMKS
              200       210       220       230       240       250       260

Tip C484-77
Conf: 222410245321058864544167642057789999999987655699999999999999999705112477 7359
Pred: CCCCCCCCCCCCCEEEECCCCCHHHHHCCHHHHHHHHHHHHHHHHHHHHHHHHHHHHHHHHHHCCCEEEEEECC
  AA: ANLGASQASTVRRHVREQNFKQLRERKANEGKIVKDLKRLEYKVNIILCLVVVILAIILLLTGLSILFIRIKS
               150       160       170       180       190       200       210

Tio
Conf: 1333467753001785311000016653202668999999999975304799999999999999999999999 98719
Pred: HHHHHHHHHHHCCCCCCCCCHHHHHHHHHHHHHHHHHHHHHHHHHHHHHHHHHHHHHHHHHHHHHHHHHHHHHHHHHC
  AA: ANLGQKQSATSKNGPQIILREATEVESQQATDGQLNHRVEKVEKKLTCVICLLIGILVLLILLFMLGFLFLLMK
          200       210       220       230       240       250       260
```
